# Development of a high specificity typing method for the detection of herpes simplex virus

**DOI:** 10.3389/fbioe.2022.955713

**Published:** 2022-08-17

**Authors:** Zhu Chen, Kaixuan Zhao, Boyu Tan, Zengrui Tong, Ziyu He, Xiaofang Luo, Lei Cai, Hanming Wang, Polly H. M. Leung, Franklin Wang-Ngai Chow, Hui Chen, Yan Deng

**Affiliations:** ^1^ Hunan Key Laboratory of Biomedical Nanomaterials and Devices, Hunan University of Technology, Zhuzhou, China; ^2^ Department of Scientific Research, Zhuzhou Hospital Affiliated to Xiangya School of Medical, Central South University, Zhuzhou, China; ^3^ College of Chemistry and Bioengineering, Hunan University of Science and Engineering, Yongzhou, China; ^4^ Guangzhou Wondfo iCubate Biotech Co. Ltd., Guangzhou, China; ^5^ Wondfo Biotech Co. Ltd., Guangzhou, China; ^6^ Department of Health Technology and Informatics, The Hong Kong Polytechnic University, Hong Kong, China

**Keywords:** HSV-1, HSV-2, orthogonal design, primer design, HSV typing

## Abstract

Herpes disease is caused by Herpes simplex virus (HSV). It has become one of the global health problems. This paper reports a method for HSV type testing. First specific primers sequence for HSV-1 and HSV-2 were selected, designed, and synthesized. Then, these amplification products were proved by sequencing and analysis. Lastly, we optimized the reaction system and PCR reaction program by orthogonal design and sensitivity testing. Results showed that the lowest concentration in HSV-type testing is about 6.67 × 10^6^ copies/ml. Moreover, the specificity of detection was very high. So, this method has very great potentials for HSV type testing in clinical practice.

## Introduction

Herpes disease, caused by Herpes Simplex Virus (HSV), has become one of the global health problems (Looker et al., 2017). HSV can cause high infant morbidity and infant mortality with mother-to-child transmission (James and Kimberlin, 2015). It includes Herpes simplex virus type 1 (HSV-1) and Herpes simplex virus type 2 (HSV-2), which are double-stranded DNA viruses belonging to the Herpesviridae family (Salvatore et al., 2019), causing persistent infection in the trigeminal or lumbosacral ganglia (Christine et al., 2016). Besides, the HSV-associated disease becomes malignant when the virus gets access to the central nervous system, which may result in herpes encephalitis (Koujah et al., 2018). HSV-1 mainly causes infection of skin other than genital organs, mucosa (oral mucosa), and organs (brain). HSV-2 mainly causes genital musoca skin infection. However, in these years, it is found that HSV-1 can be transmitted *via* sexual contact and genital herpes may also occur due to transmission of HSV-1 by oral-genital contact (Christian et al., 2019; Khan et al., 2019), which means that the line between HSV-1 and HSV-2 infection has become increasingly blurred and both viruses can infect genital epithelial cells and produce the same pathological mechanisms. And given that the latest literature reports that the HSV-2 has infected more than 700 million people worldwide, early selection of appropriate methods for accurate diagnosis of HSV is particularly important (Dropulic et al., 2019; Harfouche et al., 2021; Rice, 2021).

**FIGURE 1:**
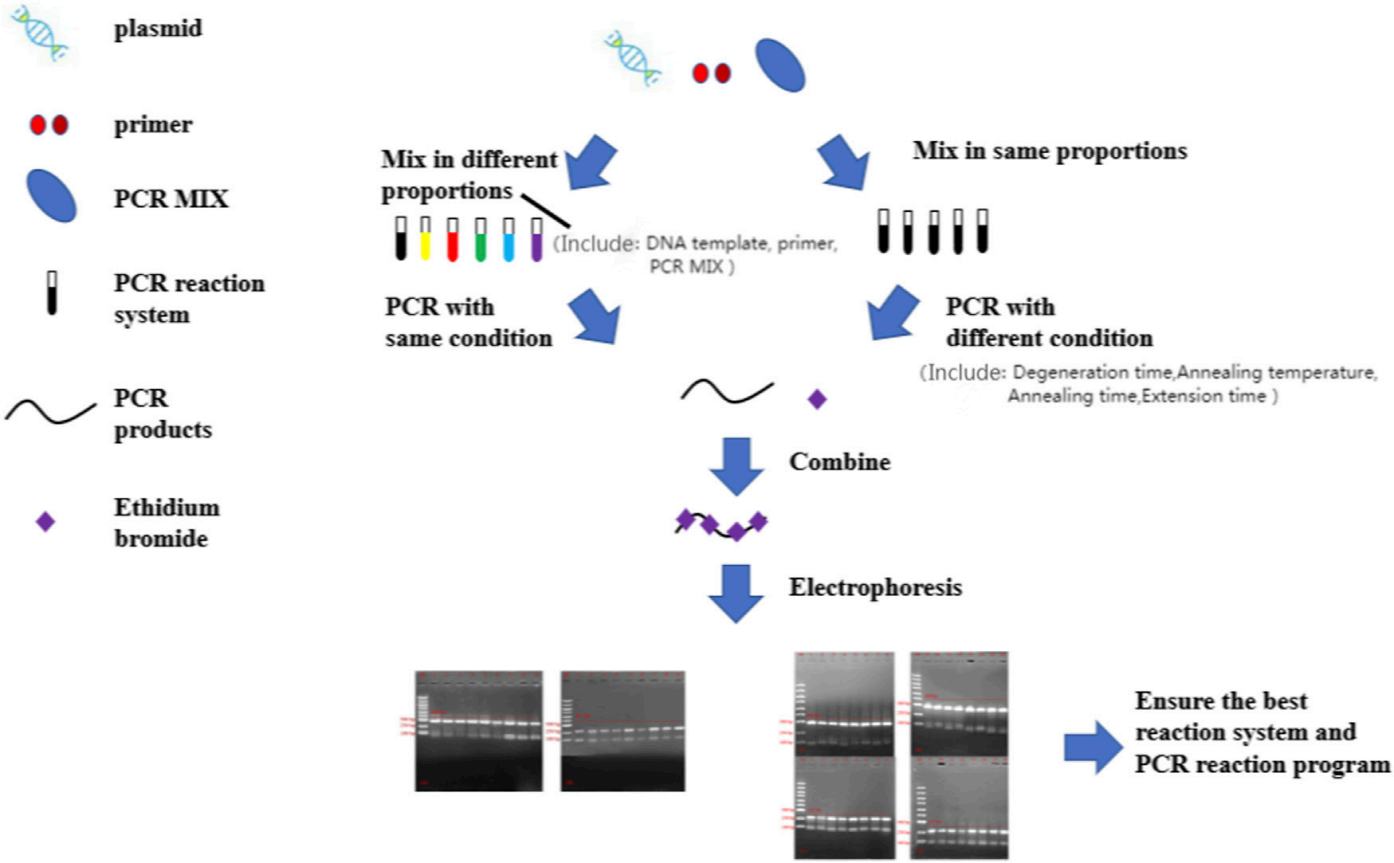
The flowchart for optimization of a high specificity typing PCR method. PCR, polymerase chain reaction.

At present, HSV detection methods mainly include cytology, immunology, molecular biology detection and so on. For example; 1) Virus isolation and culture is a reliable basis for definitive diagnosis of HSV infection in clinical practice today, but its operation is cumbersome and the time is long, and also needs other methods to assist identification (Qi et al., 2020; Wijesinghe et al., 2021). 2) Immunoassay has high specificity and insufficient sensitivity, which may lead to false negative (Chauhan et al., 2018; Fatima et al., 2021). 3) Nucleic acid detection methods in molecular biology have outstanding advantages, such as high sensitivity and specificity (Chen et al., 2020; Tang et al., 2020; Chen et al., 2022; Liu et al., 2022). So, this study aimed to design a PCR typing detection method for detecting HSV-1 or HSV-2 in the same reaction system (Figure 1), and it has high specificity and is difficult to appear with false positive. This work hopes to provide some new ideas for researchers in HSV-type identification in clinical practice.

## Materials and methods

### Materials

We chose the CDS region of HSV-1 and HSV-2 as the target gene sequence. Then we compared homologous sequences on NCBI to identify the target sequence. The plasmids from HSV-1 and plasmid from HSV-2 were synthesized by Sangon Biotech (Shanghai, China) (Table 1). The MIX used for PCR (including Taq DNA polymerase, dNTPs, buffer, etc.) was a 2x concentrated PCR amplification premixed solution purchased from Sangon Biotech (Shanghai, China). PCR not only removes the complex operation process by adding templates and primers to the system, but also saves time. According to the design principles for PCR primers, those primers were designed by using primer 5 software for our selected gene regions (Table 2).

**TABLE 1:** Sequence information for plasmid used for polymerase chain reaction.

Primer name	Sequence information (5′–3′)	Amplification length (bp)
Plasmid-HSV-1	GTC​ATT​CAG​ATA​TCC​TGT​CTG​CTC​TAC​GAC​CTG​TCC​ACC​ACC​GCC​CTG​GAG​CAC​GTC​CTC​CTG​TTT​TCG​CTC​GGT​TCC​TGC​GAC​CTC​CCC​GAA​TCC​CAC​CTG​AAC​GAG​CTG​GCG​GCC​AGG​GGC​CTG​CCC​ACG​CCC​GTG​GTT​CTG​GAA​TTC​GAC​AGC​GAA​TTC​GAG​ATG​CTG​TTG​GCC​TTC​ATG​ACC​CTT​GTG​AAA​CAG​TAC​GGC​CCC​GAG​TTC​GTG​ACC​GGG​TAC​AAC​ATC​ATC​AAC​TTC​GAC​TGG​CCC​TTC​TTG​CTG​GCC​AAG​CTG​ACG​GAC​ATT​TAC​AAG​GTC​CCC​CTG​GAC​GGG​TAC​GGC​CGC​ATG​AAC​GGC​CGG​GGC​GTG​TTT​CGC​GTG​TGG​GAC​ATA​GGC​CAG​AGC​CAC​TTC​CAG​AAG​CGC​AGC​AAG​ATA​AAG​GTG​AAC​GGC​ATG​GTG​AAC​ATC​GAC​ATG​TAC​GGG​ATT​ATA​ACC​GAC​AAG​ATC​AAG​CTC​TCG​AGC​TAC​AAG​CTC​AAC​GCC​GTG​GCC​GAA​GCC​GTC​CTG​AAG​GAC​AAG​AAG​AAG​G	490
Plasmid-HSV-2	CAG​CCC​TCG​CCC​CTG​GGT​CGG​GAG​GCG​GTG​GAA​CAG​TTC​TTC​CGG​CAC​GTG​CGC​GCC​CAG​CTG​AAC​ATC​CGC​GAG​TAC​GTA​AAG​CAA​AAC​GTC​ACC​CCC​AGG​GAA​ACC​GCC​CTG​GCG​GGA​GAC​GCG​GCC​GCC​GCC​TAC​CTG​CGC​GCG​CGC​ACG​TAT​GCC​CCG​GCG​GCC​CTC​ACG​CCC​GCC​CCC​GCG​TAC​TGC​GGG​GTC​GCA​GAC​TCG​TCC​ACC​AAA​ATG​ATG​GGA​CGT​CTG​GCG​GAA​GCA​GAA​AGG​CTC​CTA​GTC​CCC​CAC​GGC​TGG​CCC​GCG​TTC​GCA​CCA​ACA​ACC​CCC​GGG​GAC​GAC​GCG​GGG​GGC​GGC​ACT​GCC​GCC​CCC​CAG​ACC​TGC​GGA​ATC​GTC​AAG​CGC​CTC​CTC​AAG​CTG​GCC​GCC​ACG​GAG​CAG​CAG​GGC​ACG​ACG​CCC​CCG​GCG​ATC​GCG​GCT​CTC​ATG​CAG​GAC​GCG​TCG​GTC​CAA​ACC​CCC​CTG​CCC​GTG​TAC​AGG​ATT​ACC​ATG​TCC​CCG​ACC​GGC​CAG​GCG​TTT​GCC​GCG​GCG​G	490

**TABLE 2:** Sequence information of the primers used for polymerase chain reaction.

Primer name	Sequence information (5′–3′)	Amplification length (bp)
PCR-H1-F	ATA​TCC​TGT​CTG​CTC​TAC​GAC​CTG​TCC​ACC​AC	469
PCR-H1-R	TCC​TTC​AGG​ACG​GCT​TCG​GCC​ACG​GCG​TTG​AG	
PCR-H2-F	AAA​TGA​TGG​GAC​GTC​TGG​CGG​AAG​CAG​AAA​GG	267
PCR-H2-R	GCA​AAC​GCC​TGG​CCG​GTC​GGG​GAC​ATG​GTA​A	

### Design primer for PCR assays

We design the two pairs of designed primers for PCR and verify them useful. The reaction components for PCR assays were as follows: 12.5 μl PCR Mix, 10 μl dd H_2_O, 0.5 μl HSV-1 template or HSV-2 template, 1 μl primer 1-F and 1 μl primer 1-R or 1 μl primer 2-F and 1 μl primer 2-R. It was amplified in Eppendorf Master cycler PCR instrument (Eppendorf, Hamburg, Germany) for predegeneration at 72°C for 5 min, and then 35 cycles at 95°C 30 s, 58°C for 30 s, and 72°C 30 s, followed by a final extension at 72°C for 10 min. The PCR products were sequenced by Sangon Biotech (Shanghai, China), after being analyzed by 1% agarose gel.

### PCR typing assays

To verify whether the typing PCR was feasible. The reaction components for the PCR assays were as follows:12.5 μl PCR Mix, 10 μl dd H_2_O, 0.5 μl HSV-1 template or HSV-2 template, 0.5 μl primer 1-F, 0.5 μl primer 1-R, 0.5 μl primer 2-F, 0.5 μl primer 2-R. It was amplified in Eppendorf Master cycler PCR instrument (Eppendorf, Hamburg, Germany) for predegeneration at 95°C for 5 min, and then 35 cycles at 95°C 30 s, 58°C for 30 s, and 72°C 30 s, followed by a final extension at 72°C for 10 min. Finally, the PCR products were analyzed by 1% agarose gel.

### Optimization of reaction system by orthogonal design

Referring to others’ work (Zeng et al., 2015; Zeng et al., 2016; Tang et al., 2018; Mou et al., 2019; Gong et al., 2021), the gradient experiments for single factor and multilevel orthogonal design were integrated and results from the two experiments were analyzed to find the best PCR reaction system (Table 3).

**TABLE 3:** Reaction system information for optimization by orthogonal design (μl).

Number	dd H_2_O	PCR MIX	Primer 1	Primer 2	DNA
1	7.0	15.0	1.5	1.0	0.5
2	10.0	12.5	1.2	0.8	0.5
3	13.0	10.0	0.9	0.6	0.5
4	7.0	15.0	1.25	1.25	0.5
5	10.0	12.5	1.0	1.0	0.5
6	13.0	10.0	0.75	0.75	0.5
7	7.0	15.0	1.0	1.5	0.5
8	10.0	12.5	0.8	1.2	0.5
9	13.0	10.0	0.6	0.9	0.5

### Optimization of PCR reaction program by orthogonal design

As shown in Table 4, the gradient experiments for single factor and multilevel orthogonal design were integrated and results from the two experiments were analyzed to find the best PCR reaction conditions. The predegeneration was at 95°C for 5 min and final extension was at 72°C for 10 min.

**TABLE 4:** Information for polymerase chain reaction program optimization by orthogonal design.

Nos	Degeneration temperature (°C)	Degeneration time (s)	Annealing temperature (°C)	Annealing time (s)	Extension temperature (°C)	Extension time (s)
1	95	20	57	25	72	25
2	95	20	58	30	72	35
3	95	20	59	35	72	45
4	95	20	60	40	72	55
5	95	25	57	25	72	25
6	95	25	58	30	72	35
7	95	25	59	35	72	45
8	95	25	60	40	72	55
9	95	30	57	25	72	25
10	95	30	58	30	72	35
11	95	30	59	35	72	45
12	95	30	60	40	72	55
13	95	35	57	25	72	25
14	95	35	58	30	72	35
15	95	35	59	35	72	45
16	95	35	60	40	72	55

### Sensitivity experimental design

We diluted the plasmid of HSV-1 and plasmid from HSV-2 at 6.67 × 10^10^ copies/ml to 6.67 × 10^6^ copies/ml concentrations in a 10-fold gradient with dd H_2_0. Plasmids (from 6.67 × 10^8^ copies/ml to 6.67 × 10^5^ copies/ml) with different concentrations were added into the reaction system. It was then amplified in a PCR instrument (Eppendorf, Hamburg, Germany) for predegeneration at 95°C for 5 min, and then 35 cycles at 95°C 20 s, 59°C for 35 s, and 72°C 45 s, followed by a final extension at 72°C for 10 min.

## Results and discussion

### The feasibility of primers

**FIGURE 2:**
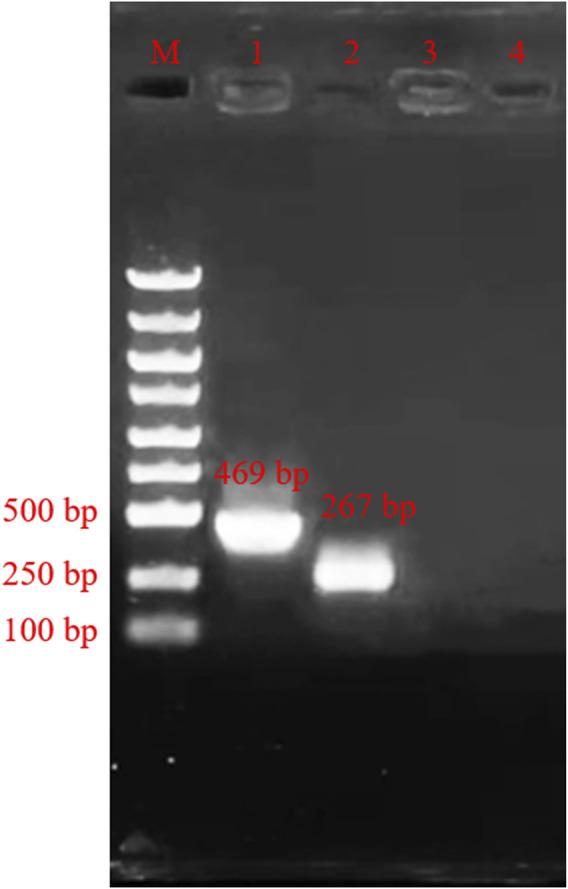
Agarose gel electrophoresis of amplification product. M: Marker; Lane 1, primers PCR-H1-F and primer PCR-H1-R; Lane 2, primers PCR-H2-F and primer PCR-H2-R; Lane 3, Negative control of primers PCR-H1-F and primer PCR-H1-R; Lane 4, Negative control of primers PCR-H2-F and primer PCR-H2-R.

**FIGURE 3:**
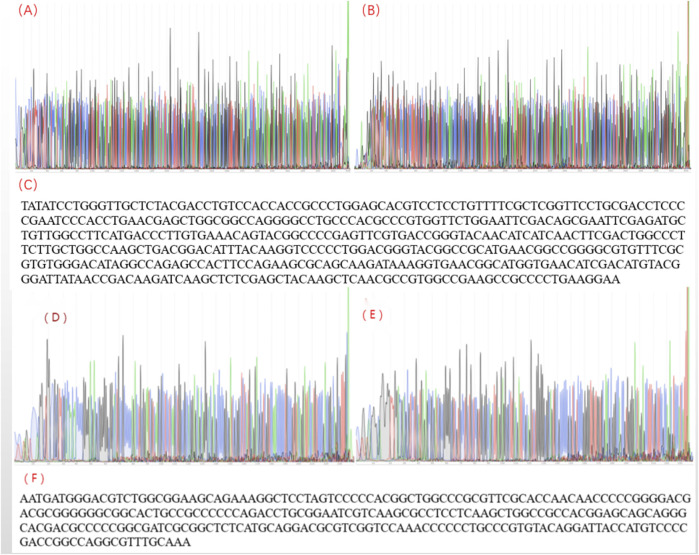
The sequencing result for herpes simplex virus polymerase chain reaction products. **(A)** The sequencing peak map for HSV-1-F; **(B)** The sequencing peak map for HSV-1-R; **(C)** Assembling from sequencing data of herpes simplex virus 1; **(D)** The sequencing peak map for HSV-2-F; **(E)** The sequencing peak map for HSV-2-R; **(F)** Assembling from sequencing data for herpes simplex virus 2.

The results from verifications of our designed primers are shown in Figure 2 below. The location of the bands was correct, and the bands had high specificity. Then the products were sent to company (Shanghai, China) for sequencing. The sequencing results are shown in Figure 3, and the results are correct. The designed primers were then used for subsequent experiments.

### PCR typing system detection

**FIGURE 4:**
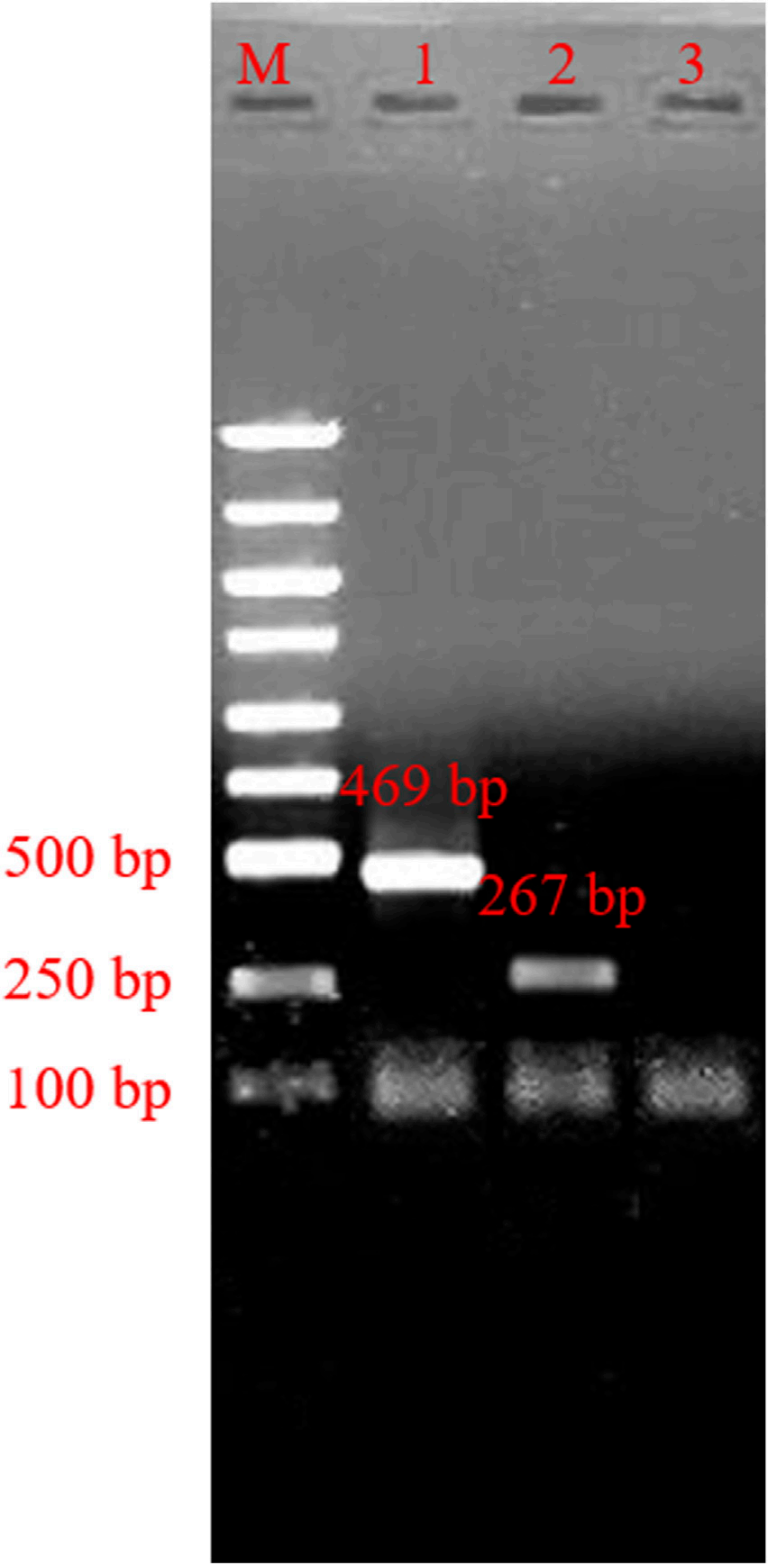
Agarose gel electrophoresis of typing polymerase chain reaction product. M: marker; line 1, typing polymerase chain reaction product of herpes simplex virus 1; line 2, typing polymerase chain reaction product of herpes simplex virus 2; Lane 3, Negative control.

Using the confirmed reaction system, we added the HSV-1 template plasmids into tube-1, and template plasmids from HSV-2 into tube-2. We then added dd H_2_O into tube-3 as a negative control. The results are shown in Figure 4. The obtained bands’ specificity was high, and nonspecific amplification did not appear in our result.

### Optimization of reaction system

**FIGURE 5:**
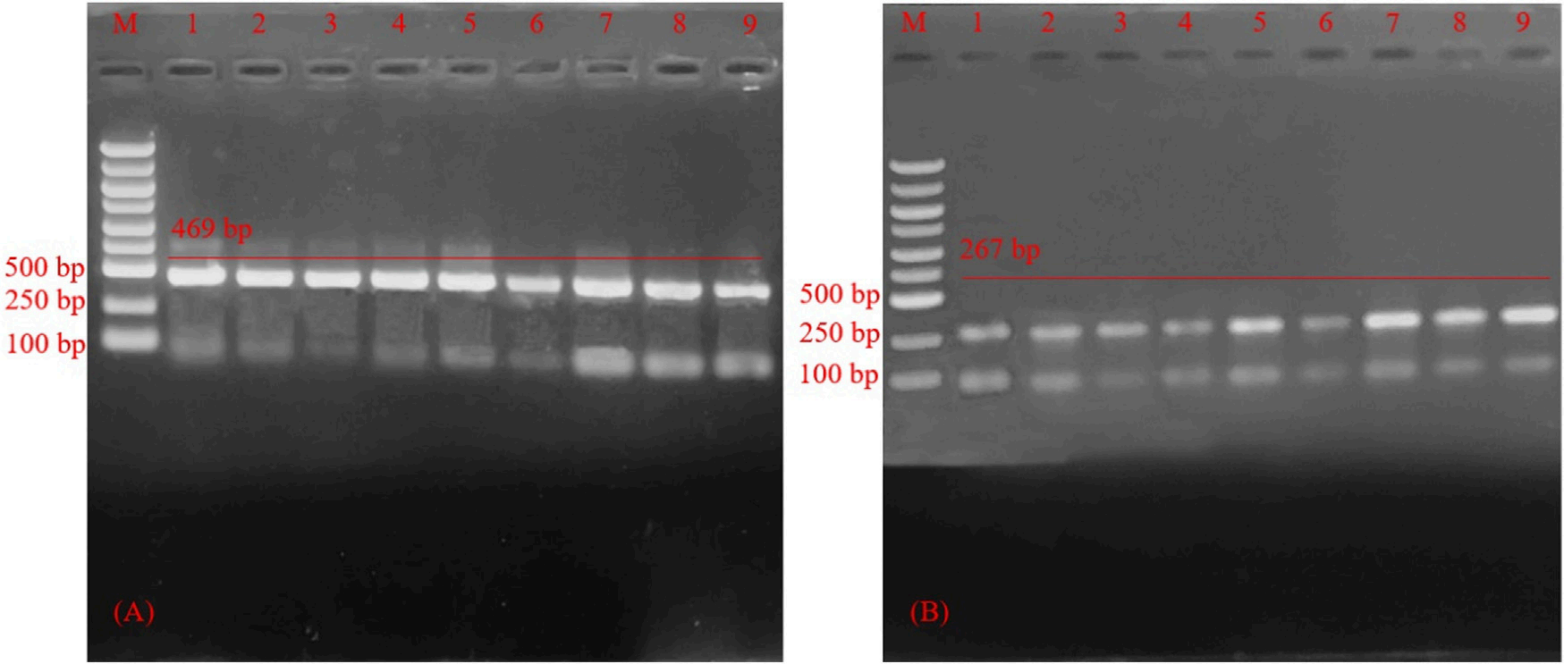
Agarose gel electrophoresis of polymerase chain reaction product of reaction system optimization **(A)** Agarose gel electrophoresis for polymerase chain reaction product of optimization of herpes simplex virus -1 reaction system. M: Marker; Lane 1–9, primers PCR-H1-F and PCR-H1-R PCR product of optimization of herpes simplex virus 1 reaction system. **(B)** Agarose gel electrophoresis for polymerase chain reaction product optimization of herpes simplex virus 2 reaction system. M: Marker; Lane 1–9, polymerase chain reaction product of optimization of herpes simplex virus 2 reaction system.

Nine different combinations of experiments were analyzed and optimized due to differences in the templates, primers, and MIX. As shown in Figure 5, we can see that 1, 2, 3, 4, 5, and 7 bands for HSV-1 were greater than 6, 8, and 9. 5, 7, 8, and 9 for the nine HSV-2 systems were significantly brighter than for other systems. Combining above analysis, system 5 (12.5 μl PCR Mix, 10 μl dd H_2_O, 0.5 μl HSV-1 template or HSV-2 template, 0.5 μl primer 1-F, 0.5 μl primer 1-R, 0.5 μl primer 2-F, 0.5 μl primer 2-R) was the best choice.

### Optimization of the PCR reaction program

**FIGURE 6:**
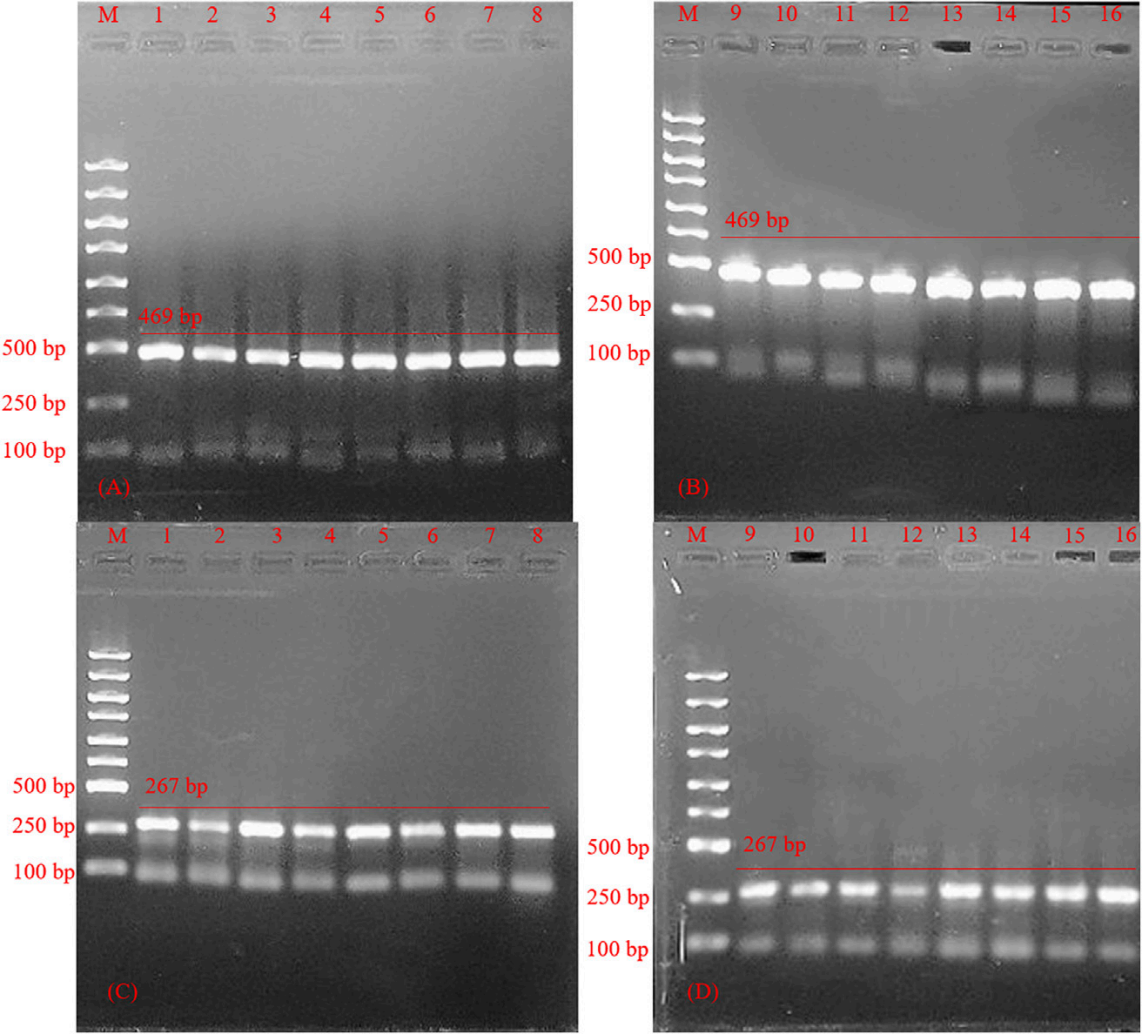
Agarose gel electrophoresis for polymerase chain reaction product optimization of polymerase chain reaction program. **(A,B)** Product of herpes simplex virus 1; M: marker; line 1–16, PCR product optimization of polymerase chain reaction program for herpes simplex virus 1; **(C,D)** Product of herpes simplex virus 2; M: marker; line 1–16, polymerase chain reaction product optimization of polymerase chain reaction program for herpes simplex virus 2.

Different combinations of 16 sets of PCR reaction programs were analyzed and optimized, and the results are shown in Figure 6. In HSV-2’s program optimization, the third program band was brighter than in the other programs. With HSV-1’s program optimization, the difference in the results was small. Combining both analyses, the third program band was clear, and had good stability. So, program 3 (degeneration time 20 s, annealing temperature 59 degrees, annealing time 35 s, extension time 45 s) was the best reaction program.

### Sensitivity inspection

**FIGURE 7:**
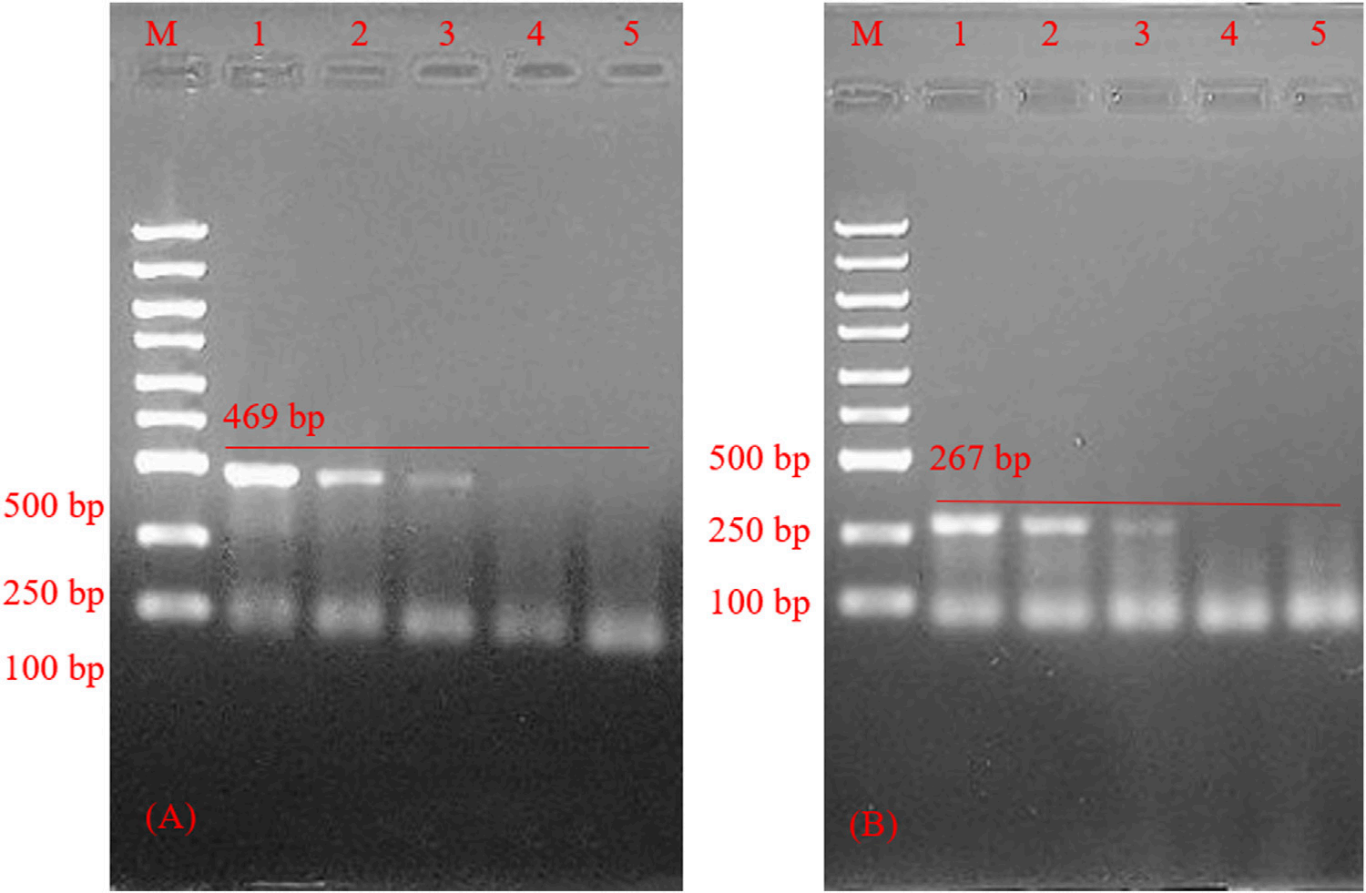
Agarose gel electrophoresis of polymerase chain reaction product sensitivity inspection. **(A)** Sensitivity inspection of herpes simplex virus 1. polymerase chain reaction product optimization of herpes simplex virus 1 reaction system; M: marker; line 1–4, Sensitivity inspection of herpes simplex virus 1 reaction system; line 5, negative control. **(B)** Sensitivity inspection of herpes simplex virus 2. M: marker; line 1–4, Sensitivity inspection of herpes simplex virus 1 reaction system; line 5, negative control.

The primer length designed in this experiment was more than 30 bp, slightly larger than the traditional PCR primer design length (The traditional PCR primer design length is about 15–30 bp). It enhanced the specificity of PCR amplification, leading to a certain decrease in the amplification efficiency as shown by results in Figure 7. 6.67 × 10^8^ copies/ml-6.67 × 10^6^ copies/ml can get bands, 6.67 × 10^5^ copies/ml can’t get bands, so the lowest concentration in HSV-type testing is about 6.67 × 10^6^ copies/ml.

## Conclusion

The current study on HSV detection was based on the HSV typing PCR reaction system and optimization of reaction procedures. Since the line between HSV-1 and HSV-2 infection has become increasingly blurred, it was necessary to establish a PCR reaction system suitable for HSV typing detection. The system is influenced by multiple aspects, including interactions of DNA concentration, primers concentration, and 2 × Taq PCR Master Mix content. This experiment is slightly different from the establishment and optimization of traditional PCR reaction system (Yang et al., 2020). The traditional PCR reaction system is a single-factor investigation method to determine the content of various components in the system. Although the herein results are intuitive, it ignores the integrity of the system (the mutual influence and internal connection between each component). The experiment adopts the orthogonal optimization method, which makes up for the deficiency of single-factor investigation, takes into account the intrinsic correlation of components, shortens the time to optimize the experimental method and saves energy. The establishment and optimization of traditional PCR reaction system usually examines the influence of five factors on the system, including (DNA template, primer, Tap DNA primer, polymerase, Mg^2+^, dNTP), this experiment adopted 2 × Taq PCR Master Mix, to transform the original complex 5 factors into 3 factors, simplifying the establishment and optimization of the reaction system and facilitating subsequent experiments. This HSV typing detection system with high availability of HSV with high stability was verified by suitable target genes and designed primers.

## Data Availability

The original contributions presented in the study are included in the article/Supplementary Materials, further inquiries can be directed to the corresponding authors.
